# Novel Treatment Modality for Chimeric Antigen Receptor T-cell Therapy Complications: A Case Report

**DOI:** 10.7759/cureus.65497

**Published:** 2024-07-27

**Authors:** Dianella Rente Lavastida, Samantha De Filippis, Eliu G Rivera Torres, Alexander Aldanese, Samir Ruxmohan

**Affiliations:** 1 Clinical Sciences, St. George's University School of Medicine, St. George's, GRD; 2 Clinical Sciences, University of Medicine and Health Sciences, Camps, KNA; 3 Clinical Sciences, St. George’s University School of Medicine, St. George's, GRD; 4 Neurocritical Care, University of Texas (UT) Southwestern Medical Center, Dallas, USA

**Keywords:** tocilizumab, blinatumomab, neurooncology, neurotoxicity, neurocritical care

## Abstract

Immune cell-associated neurotoxicity syndrome (ICANS) and cytokine release syndrome (CRS) are both common adverse effects of chimeric antigen receptor (CAR) T-cell therapy. Blinatumomab is a commonly used CAR T-cell treatment in patients with B-cell acute lymphoblastic leukemia (B-ALL). Our patient presented with an extensive past medical history, including refractory B-ALL, and developed CRS and ICANS following treatment with blinatumomab CAR-T cell therapy. Early clinical detection of ICANS, monitoring using immune effector cell encephalopathy scores, following the appropriate protocol for ICANS grade, and adding anakinra (IL-1 receptor antagonist) were crucial steps in managing his condition. The approach to managing and monitoring this patient was unique in that we added anakinra to the standard treatment regimen. With this report, we emphasize the need for further research regarding CAR T-cell therapeutic regimens and how to decrease the morbidity and mortality of its adverse effects.

## Introduction

Chimeric antigen receptor (CAR) T-cell therapy is a recent therapeutic innovation currently used to target hematologic malignancies, where a patient's own T-cells are engineered to express specific antigen receptors, which then identify and attack cells with that specific target antigen [1]. Some of the CAR T-cell therapy’s limitations include its associated toxicities, the most common of these being immune cell-associated neurotoxicity syndrome (ICANS) and cytokine release syndrome (CRS) [2]. 

CRS is defined as the release of inflammatory cytokines like IL-6, IL-10, IFN, and TNF-α by activated lymphocytes, myeloid cells, or even non-immune cells [3]. While the specific cytokines released vary, CRS symptoms triggered by acute inflammatory responses typically occur 1 to 14 days after CAR T-cell infusion and commonly include fever, tachycardia, malaise, and fatigue in conjunction with elevated lactate dehydrogenase, C-reactive protein, and ferritin. It may also lead to more severe symptoms like hypotension, respiratory insufficiency, acute kidney injury, and end-organ damage [4,5]. 

ICANS is a common adverse effect of CAR T-cell therapy, occurring in 20-70% of patients [2]. ICANS commonly occurs within a week of infusion; however, in about 10% of patients, it has been shown to present up to three weeks after CAR T-cell infusion; it can also occur immediately after or during CRS [2]. ICANS is typically reversible and lasts 2-4 days [2]. Although the underlying mechanism behind ICANS is somewhat obscured, it is believed to be caused by the passive diffusion of cytokines into the brain and/or trafficking of T-cells into the central nervous system (CNS), which, in turn, increases vascular permeability and endothelial activation, leading to blood-brain barrier breakdown [1]. ICANS is a clinical diagnosis, and early symptoms include word-finding difficulties, confusion, impaired fine motor skills, hesitancy of speech, and deterioration in handwriting due to the development of tremors. These symptoms can progress to aphasia with both expressive and receptive components. Signs and symptoms associated with higher mortality include status epilepticus, cerebral edema, or intracerebral hemorrhage [6].

It is recommended for patients with suspected deterioration to be assessed twice daily using the immune effector cell encephalopathy (ICE) score [7]. This score can then be integrated into the complete assessment to obtain an ICANS grade. A higher ICE score leads to a lower ICANS grade. Patients with an ICE score less than 2 or with seizures are classified as severe (grades 3 and 4) and should be transferred to an intensive care unit (ICU). Factors associated with higher risk of grade 3 ICANS include higher disease affliction, thrombocytopenia, and the potential to develop severe CRS. It is important to note that with supportive care and early intervention, most cases will resolve. Management of ICANS is dependent on the severity of the ICE score and ICANS grade, as well as the occurrence of CRS [8].

Of note, anakinra was added to this patient’s therapy. Anakinra is an IL-1R antagonist that is FDA-approved for disorders such as rheumatoid arthritis and has been suggested as a third-line agent for refractory CAR T cell-associated toxicities by the Society for Immunotherapy of Cancer [9].

## Case presentation

We present the case of a 54-year-old male with a past medical history of HIV, type 2 diabetes mellitus, coronary artery disease, chronic kidney disease, and relapsed B-cell acute lymphocytic leukemia (B-ALL). Medications he was taking at home included highly active antiretroviral therapy (HAART- emtricitabine-tenofovir alafenamide), acyclovir, albuterol sulfate, benzonatate, bupropion, dextromethorphan-guaifenesin, esomeprazole, fluconazole, gabapentin, guaifenesin SR, levofloxacin, levothyroxine, metoprolol tartrate, montelukast, ondansetron, and oxycodone-acetaminophen. 

The patient was admitted to the hospital on March 6th, 2024, and was started on cycle 1 of IV blinatumomab at a rate of 28 mcg daily for relapse of B-ALL two days later, after which he developed fever and tachycardia that same day. The patient received tocilizumab for CRS three days after admission and began to exhibit sudden altered mental status and suspected seizures on day four. At this point, administration of blinatumomab was stopped, and ICE score checks (Table 1) began and remained 10/10 until day four at approximately 23:00, when there was a sudden mental status change, revealing an ICE score of 0/10. Seven days after admission, he was transferred to the Neurological ICU due to worsening mental status, where a lumbar puncture was performed to rule out infectious encephalopathy. After consideration of multiple differentials, the etiology was narrowed down to blinatumomab toxicity. 

**Table 1:** ICE score breakdown This table is adapted from "Chapter 27: Management of Immune Effector Cell-Associated Neurotoxicity Syndrome (ICANS)" [10], licensed under CC BY-NC 4.0 (https://creativecommons.org/licenses/by-nc-nd/4.0/).

Immune Effector Cell Encephalopathy (ICE) Score		
Assessment of Cognition		Total Points
Orientation	Orientation to year, month, city, hospital	4 points
Naming	Ability to name 3 objects (e.g., point to clock, pen, button)	3 points
Following commands	Ability to follow simple commands (e.g., “show me 2 fingers” or “close your eyes and stick out your tongue”)	1 point
Writing	Ability to write a standard sentence (e.g., “our national bird is the bald eagle”)	1 point
Attention	Ability to count backward from 100 by 10	1 point

**Figure 1:**
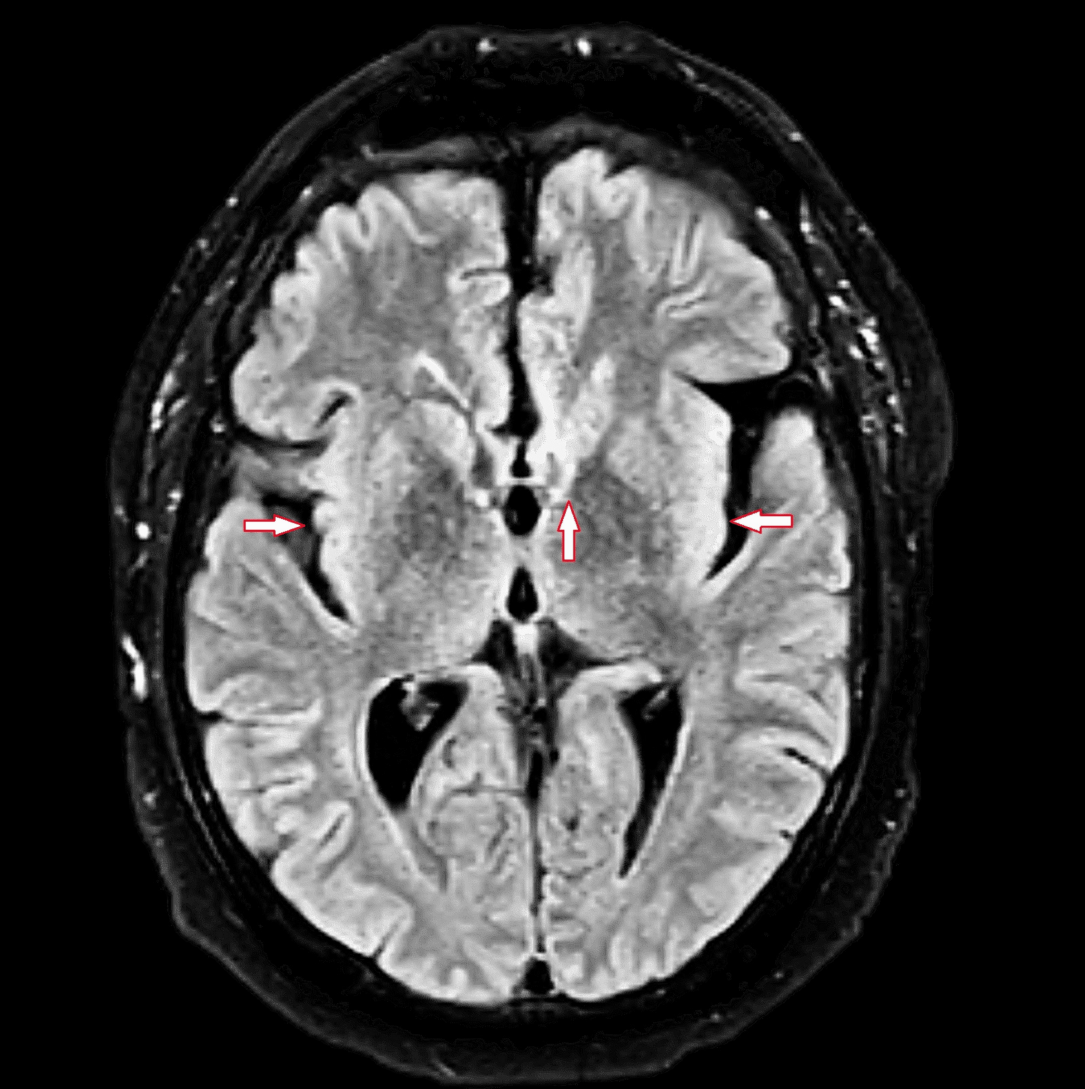
T2 FLAIR MRI of brain without contrast showing scattered foci of FLAIR hyperintensity at the white matter of both cerebral hemispheres, nonspecific, likely representing chronic microvascular ischemic changes. FLAIR: Fluid attenuated inversion recovery

**Figure 2:**
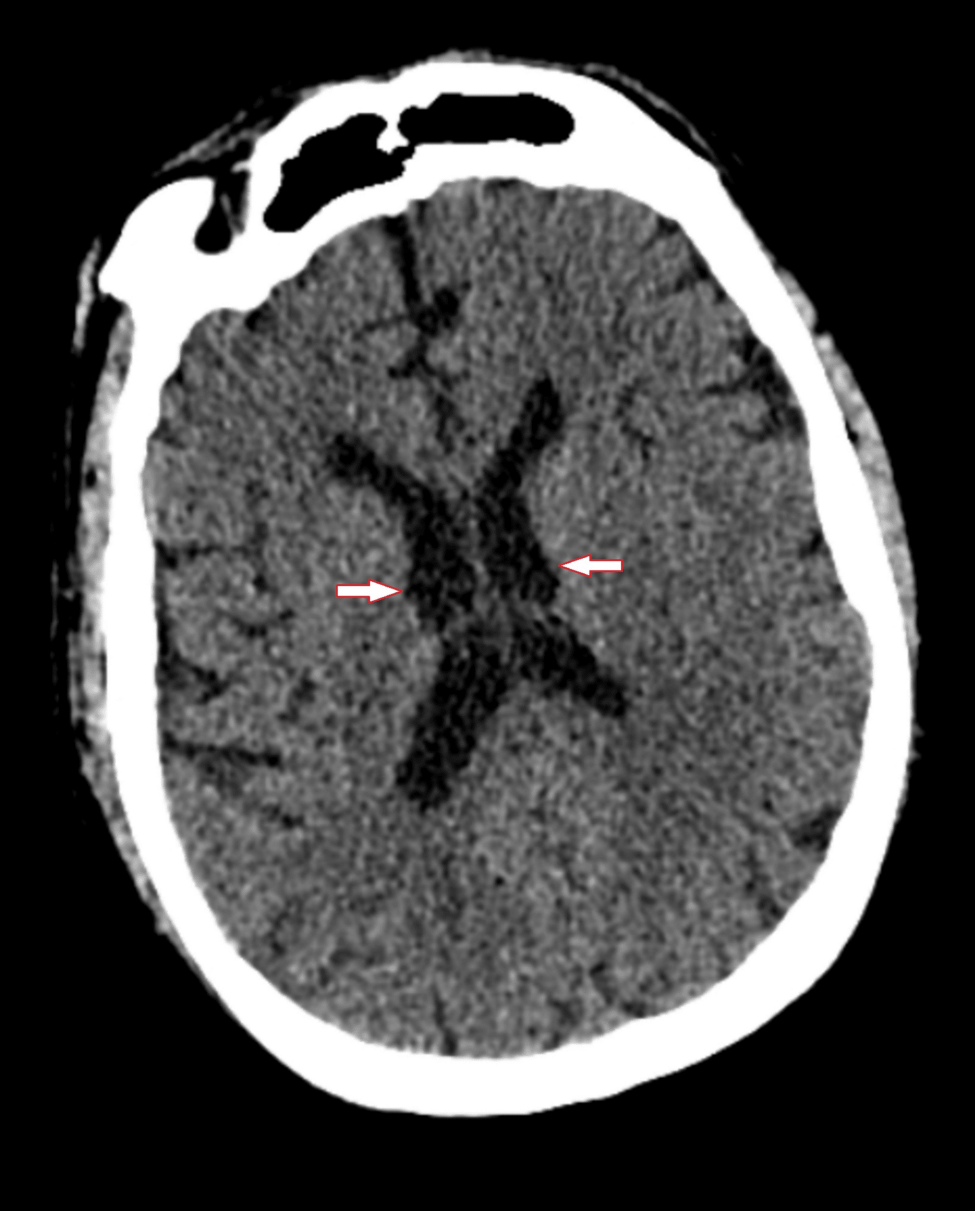
CT of brain without contrast showing lateral ventricles are normal in size and configuration. No hydrocephalus, midline shift, or extra-axial fluid collection.

In the ICU, the patient was emergently started on IV dexamethasone 20 mg q8h, IV levetiracetam 750 mg q12h, Anakinra 200 mg TID, and Precedex drip for agitation. A plan was established to perform q1h neuro checks and ICE score checks. Additionally, a brain MRI (Figure 1), CT of the head (Figure 2), and electroencephalogram (EEG) recording were obtained. The initial physical exam in the ICU revealed an unresponsive and stuporous patient with no attempted speech who resisted eye-opening, and the patient was lying on his side while fighting the examination procedure without clear focality, yielding a very limited exam. He was still able to withdraw all extremities to noxious stimuli. The patient was a poor historian, and there were no family members at the bedside at that time. 

Our patient eventually improved after extensive ICU treatment and monitoring, returning to his baseline of full alertness and orientation. He was then transferred back to the cancer floor, and recommendations for follow-up outpatient were made.

## Discussion

This case discusses a patient who developed ICANS shortly after beginning CARS T-cell therapy. Our patient’s disease reached a grade of 3, which was indicative of immediate ICU management. Though ICANS typically resolves within 2-4 days after diagnosis, early management and intervention may decrease the risk of mortality and morbidity [2]. 

ICANS may present biphasically, with the first phase occurring concurrently with fever and CRS within five days of T-cell infusion, and typically has a lower ICE grade. The second phase occurs after the fever and CRS subsides and commonly has a higher ICE score and may last longer [2]. Monitoring ICANS with frequent ICE scores is imperative to catching this complication early and should be a part of CAR T-cell treatment assessment. After the establishment of ICANS grade, patients are able to be treated accordingly. The grading scale can be reviewed in Table 2. 

**Table 2:** Comprehensive breakdown of CRS and ICANS symptoms with management steps This table is adapted from "How I treat adverse effects of CAR-T cell therapy" [11], licensed under CC BY-NC 4.0 (https://creativecommons.org/licenses/by-nc-nd/4.0/). CRS: Cytokine release syndrome; ICANS: immune cell-associated neurotoxicity syndrome

Cytokine Release Syndrome						Immune Effector Cell-Associated Neurotoxicity Syndrome					
ICU	Therapy	Hypoxia	Low BP	Fever ≥38⁰C	Grade	ICE score	Alertness	Seizure	Cerebral Edema	Therapy	ICU
Unnecessary	If 3 days of Grade 1→ consider Tocilizumab Tx	Absent	Absent	Present	1	7-9	Spontaneous awakening	Absent	None	Monitor, supportive	Notify ICU & Neurologist
Notify ICU	Tocilizumab Tx	If present→ O_2_ supplement ≤61/min	Present→ no vasopressors	Present	2	3-6	Awakens to voice	Absent	None	Dexamethasone, if CRS ≥1→ add tocilizumab	Notify ICU & Neurologist
ICU Management	Tocilizumab & dexamethasone Tx	If present→ O_2_ supplement >61/min	Present→ 1 vasopressor	Present	3	0-2	Awakens to tactile stimulus	Focal or generalized but not reflected on EEG (fast resolution)	Local edema on imaging without bleeding	Dexamethasone; if CRS ≥1→ add Tocilizumab	ICU management
ICU Management	Tocilizumab, dexamethasone, mechanical ventilation, & High dose methylprednisolone	If present→ positive pressure with CPAP, BPAP, Mechanical vent	Present→ requires two or more vasopressors (not vasopressin)	Present	4	Unable to obtain ICE score	Unarousable or requires vigorous stimuli	Status epilepticus (≥5 mins) or repetitive electric seizure without return to normal	Diffuse edema on imaging; decerebrate or decorticate posture; CN 4 palsy or Cushing Triad	High-dose methylprednisolone; if CRS ≥1→ add Tocilizumab	ICU management

Grade 1 ICANS has ICE scores of 7-9 and presents with mild neurocognitive impairment. Management of this condition requires vigilant supportive care, aspiration precautions, and intravenous hydration. In patients with cognitive impairment, it is critical to avoid medications that induce CNS depression. However, for agitated patients, low doses of lorazepam or haloperidol may be administered with careful monitoring. It is also recommended to place a neurology consultation and to perform a fundoscopic examination to assess for papilledema. Patients may benefit from neuroimaging with an MRI of the brain, both with and without contrast. CT scan is an acceptable alternative if MRI is not feasible. Diagnostic lumbar punctures should be performed, and an MRI of the spine should be considered if the patient presents with focal peripheral neurological deficits. Daily 30-minute EEGs are suggested until symptoms of toxicity resolve. Administration of levetiracetam 750 mg every 12 hours may be beneficial. Additionally, tocilizumab (anti-IL6) 8 mg/kg IV should be considered if ICANS is accompanied by concurrent CRS [6].

Grade 2 ICANS has ICE scores of 3-6 and commonly presents with moderate neurocognitive impairment. Management of this condition includes supportive care and a neurological workup as described for grade 1. Tocilizumab 8 mg/kg IV is recommended if the condition is associated with concurrent CRS. For cases refractory to tocilizumab therapy or ICANS without concurrent CRS, dexamethasone 10mg IV every 12 hours should be administered. ICU care should be considered when ICANS is associated with CRS of grade 2 or higher [10,11].

Grade 3 ICANS has ICE scores 0-2 and presents with severe neurocognitive impairment. It also includes stage 1-2 papilledema or cerebral spinal fluid (CSF) opening pressure <20 mmHg and partial seizures or nonconvulsive seizures on EEG that respond to benzodiazepines. Management of grade 3 includes supportive care and a neurological workup similar to grade 1. ICU transfer is recommended for patients with severe symptoms. Tocilizumab therapy should be initiated if ICANS is associated with concurrent CRS, as described for grade 2 ICANS, provided it has not been administered previously. If symptoms worsen despite tocilizumab therapy, or in cases of ICANS without concurrent CRS, dexamethasone 20 mg IV every six hours should be given, continuing corticosteroids until improvement to grade 1 ICANS is observed, followed by a tapering regimen. For patients with persistent grade 3 or higher ICANS, repeat neuroimaging (CT or MRI) every 2-3 days should be considered [10,11].

ICE scores are generally unobtainable for grade 4 ICANS and are considered life-threatening. Grade 4 ICANS is associated with stage 3-5 papilledema, CSF opening pressure >20 mmHg, or cerebral edema, as well as generalized seizures or status epilepticus. Management of grade 4 ICANS includes supportive care and a thorough neurological workup. Patients should be monitored in the ICU, with consideration given to mechanical ventilation for airway protection, if necessary. Tocilizumab and repeat neuroimaging should be conducted as outlined for grade 3 ICANS. High-dose corticosteroids should be administered and continued until ICANS symptoms improve to grade 1, followed by a tapering regimen. An example regimen includes methylprednisolone IV 1g/day for three days, followed by a rapid taper at 250 mg every 12 hours for two days, 125 mg every 12 hours for two days, and 60 mg every 12 hours for two days. Stage 3 or higher papilledema, with a CSF opening pressure of 20 mmHg or more, or the presence of cerebral edema should be managed as per the relevant treatment algorithm discussed later in this section [5,10,11].

In patients with ICANS, management of convulsive status epilepticus involves assessing the patient's airway, breathing, circulation, and blood glucose levels. In addition to being transferred to the ICU, initial treatment includes administering lorazepam 2mg IV, with an additional 2 mg IV if needed, up to a total of 4 mg, to control seizures. This should be followed by a levetiracetam 500 mg IV bolus and subsequent maintenance doses. If seizures persist, phenobarbital should be added at a loading dose of 15 mg/kg IV. Maintenance therapy after seizure control includes lorazepam 0.5 mg IV every eight hours for three doses, levetiracetam 1,000 mg IV every 12 hours, and phenobarbital 1-3 mg/kg IV every 12 hours. Continuous EEG should be in place if seizures remain refractory to treatment [10]. 

In these patients, management of papilledema varies based on the stage and associated cerebral edema. For stage 1 or 2 papilledema with a CSF opening pressure of <20 mmHg and without cerebral edema, acetazolamide is recommended. The loading dose is 1,000 mg IV, followed by maintenance doses of 250-1,000 mg IV every 12 hours, adjusted based on renal function and acid-base balance, which should be monitored 1-2 times daily. Stage 3, 4, or 5 papilledema with any sign of cerebral edema on imaging or a CSF opening pressure of 20 mmHg or more, high-dose corticosteroids with methylprednisolone IV 1 g/day, as recommended for grade 4 CAR T cell-related encephalopathy syndrome, should be used. It is ideal for the head of the bed to be elevated at 30 degrees and to place the patient on hyperventilation for a target partial pressure of arterial carbon dioxide (PaCO2) of 28-30 mmHg, maintained for no longer than 24 hours. When using hyperosmolar therapy, mannitol (20 g/dl solution) or hypertonic saline (3% or 23.4%) can be utilized. The initial dose of mannitol is 0.5-1 g/kg, with maintenance doses of 0.25-1 g/kg every six hours. The metabolic profile and serum osmolality should be monitored every six hours, withholding mannitol if serum osmolality is 320 mOsm/kg or higher or if the osmolality gap is 40 or more. For hypertonic saline, the initial dose is 250mL of 3% hypertonic saline, with a maintenance dose of 50-75 ml/h. Electrolytes should be monitored every four hours, and the infusion should be withheld if serum sodium levels reach 155 mEq/L or higher. For patients with imminent herniation, an initial 30 mL of 23.4% hypertonic saline should be administered, repeating after 15 minutes if needed [10].

In the case of patients with an Ommaya reservoir, an intraventricular catheter, CSF should be drained to achieve a target opening pressure of less than 20 mmHg. Consideration should be given to a neurosurgery consultation and the use of IV anesthetics for a burst-suppression pattern on EEG. Metabolic profiling should be performed every 6 hours, with daily head CT scans. Adjustments in medications are necessary to prevent rebound cerebral edema, renal failure, electrolyte abnormalities, hypovolemia, and hypotension [10].

Our patient with grade 3 ICANS was given IV dexamethasone 20 mg every eight hours, IV levetiracetam 750 mg every 12 hours, Anakinra 200 mg every eight hours, and precedex drip for agitation after discontinuing blinatumomab. Imaging was ordered, and EEG was performed due to high suspicion of seizures. This case followed the standard of care for patients with grade 3 ICANS but with the addition of anakinra, an IL-1 antagonist. Anakinra has been found to improve indices of inflammation and modulate cytokine levels in patients who developed corticosteroid-refractory ICANS after treatment with CD19 CAR T-cell therapy [9]. Anakinra has also been shown to be safe without increasing the risk of infections [9,12]. Though this medication is typically reserved for ICANS refractory to corticosteroids, our case suggests that initial treatment with anakinra may yield efficacious results and aid in the resolution of symptoms safely due to its relatively low risk of adverse events [13].

## Conclusions

CAR T-cell therapy is an innovative and interactive therapeutic approach that is able to target tumors using T cells that are programmed to bypass barriers that other methods of cancer treatment face. Although revolutionary in the realm of immunotherapy, some common and potentially detrimental effects include ICANS and CRS. This article explores the case of a 54-year-old male with a past medical history of HIV, type 2 diabetes mellitus, coronary artery disease, chronic kidney disease, and relapsed B-ALL. He was on blinatumomab CAR-T cell therapy from which he developed CRS, requiring treatment with tocilizumab. Subsequently, the patient suffered a cognitive decline and was transferred to the ICU. Ultimately, our patient was treated with the standard of care for grade 3 ICANS plus the addition of anakinra (anti-IL-1). A limitation of our study may be the fact that the patient was a poor historian upon admission to the ICU and there was no family at the bedside, limiting the amount of relevant and accurate information we could gather about the patient. This report emphasizes the importance of early detection and monitoring of ICANS using ICE scores and highlights the risks associated with CAR-T cell therapy. The unique treatment method used to stabilize this patient emphasizes the need for further studies to be done on the treatment regimen of ICANS.
